# Enhancing Maritime Safety Through Needs Analysis: Identifying Critical English Communication Skills for Pre-Service Maritime Students in a Chinese University

**DOI:** 10.3390/bs16010130

**Published:** 2026-01-16

**Authors:** Xingrong Guo, Mengyuan Zhen, Yiming Guo

**Affiliations:** 1 College of Foreign Languages, Shanghai Maritime University, 1550 Haigang Ave, Shanghai 201306, China; 202230810003@stu.shmtu.edu.cn; 2 School of Economics and Management, Shanghai Maritime University, 1550 Haigang Ave, Shanghai 201306, China

**Keywords:** learner profiling, maritime English, needs analysis, Chinese seafarers, maritime safety

## Abstract

Effective communication in English is a critical behavioral competency for seafarers in a multilingual maritime environment, directly impacting operational safety. However, a gap exists between current Maritime English (ME) training in China and the actual communication demands of global seafaring. This study aims to identify the specific ME skills including linguistic, behavioral, and sociolinguistic dimensions that are most important for on-board performance and safety management from the perspective of pre-service maritime students at Shanghai Maritime University. A mixed-methods approach was used, combining structured questionnaires (n = 313) with in-depth follow-up interviews (n = 10). The results identified 24 highly needed ME skills, particularly focused on areas governing safety-critical behaviors, such as wireless communication, security protocols, and emergency procedures. In addition, based on learner profiling, the study depicts two different learner characteristics: exam-focused and work-focused students, each with different views on the importance of skills. Work-focused students place greater emphasis on the practicality of their skills. The interview data confirms and enriches these quantitative research results. The research findings emphasize that ME courses must be more closely aligned with real-world communicative scenarios and behaviors, prioritize scenario based teaching and practical operations, and tailor differentiated teaching based on learner psychology and behavioral preference. This study offers references for maritime education institutions with similar learner profiles to optimize ME curricula, prioritize secure communication skills, and strengthen industry-education collaboration, thereby enhancing pre-service maritime students’ safety behavior and professional competitiveness in China.

## 1. Introduction

As the designated working language under the International Maritime Organization (IMO)’s Convention on Standards of Training, Certification and Watchkeeping for Seafarers (STCW), Maritime English (ME) is crucial for effective communication, operational efficiency, and safety in international shipping. Seafarers and ship managers are required to master basic ME to ensure compliance and risk mitigation in routine and emergency scenarios (International Maritime Organization [IMO], 2015). Maritime safety is inherently dependent on precise linguistic communication, and language barriers or communicative misunderstandings in shipboard interactions constitute critical safety risks that may trigger operational incidents (Gabedava & Hu, 2025).

Classified as a specialized sub-genre of English for Specific Purposes (ESP), ME’s role in safeguarding maritime operations has been underscored by recent empirical research (Gabedava & Hu, 2025). Yin et al. (2025) conducted a comprehensive analysis of Voyage Data Recorder (VDR) communication logs from 429 maritime accidents and near-miss incidents (2018–2023), revealing that miscommunication stemming from speech act ambiguities and lexical imprecision contributes to 37.77% of accidents, with 62.33% of near-misses and high-risk situations linked to similar linguistic gaps. Their findings emphasize the imperative of enhancing speech act clarity, lexical accuracy, and adherence to Standard Marine Communication Phrases (SMCP) in ME instruction. Inadequate ME proficiency can lead to misunderstandings, navigational errors, and legal liabilities, while international maritime law further mandates that crew members demonstrate ME competence to meet statutory requirements during international voyages.

The standardized use of ME can help reduce the risk of accidents. Maritime communication errors are a major contributing factor to maritime accidents, and inadequate English communication constitutes a significant safety risk in such scenarios (Fan et al., 2017). From a behavioral science perspective, such communication failures often stem from gaps between linguistic competence and behavioral performance in high-stakes environments, as highlighted by Reason’s model of human error (Reason, 1990), which emphasizes the interaction between individual skills, organizational factors, and system design in shaping safety outcomes.

ME is highly standardized and technical, requiring users to master both basic English language skills and specific maritime knowledge. With the continuous growth of the number of Chinese seafarers (Zhang & Zhao, 2015), ME has become increasingly critical for ensuring operational efficiency and crew safety. However, the ME proficiency of Chinese seafarers is often unsatisfactory. Some research has shown that many Chinese seafarers regarded English communication as a major challenge (Fan et al., 2017). This language barrier is exacerbated by cultural differences in communication styles, thinking patterns, and expression habits, which often lead to misunderstandings between Chinese sailors and foreign colleagues. This underscores the need to integrate cross-cultural communication theories into ME training. Guo et al. (2025) suggest that streamlining and standardizing navigation communication procedures will contribute to more effective on-board operations.

To address these challenges and help improve the efficiency of current ME teaching, it is necessary to strengthen the practicality and pertinence of ME training. As suggested by Brown (2016), needs analysis is the foundation for developing effective language courses. It is necessary to align ME instruction with behavioral science frameworks, such as Crew Resource Management (CRM), which emphasizes effective communication, situational awareness, and teamwork as core competencies for reducing human error. It helps to enhance the transfer of linguistic skills to real-world performance.

This study aims to conduct a systematic needs analysis of pre-service Chinese maritime students at Shanghai Maritime University, identifying the most important ME skills required in real-world maritime contexts and investigating how learner characteristics influence these needs. The findings of this study are expected to inform the design of more targeted and effective ME instruction for pre-service maritime students in similar university contexts, ultimately improving their communicative competence and operational safety. By identifying the most critical communication skills and the different levels of learners’ needs, this study seeks to offer evidence-based references for optimizing ME curriculum design in institutions with comparable learner profiles.

## 2. Theoretical Framework and Literature Review

ME plays an essential role in the global shipping industry. It serves as the foundation for international exchange, a guarantee for shipping safety and legal compliance, and a key enabler for career development and technological progress. However, Fan et al. (2017) found that Chinese maritime students and seafarers generally lack sufficient English communicative competence, due to factors such as the ME exam system, teaching materials, teaching methods, and students’ own limitations. Even after completing relevant courses, many maritime students’ actual ME proficiency often fails to meet industry requirements. Strengthening research on ME education and enhancing the practitioners’ language proficiency are important tasks for the future development of the maritime industry.

### 2.1. Theoretical Foundations

To address the conceptual gap between ME skills and behavioral performance, this study draws on the following key theoretical frameworks: Crew Resource Management (CRM), Reason’s model of human error, and ESP needs analysis theory.

CRM, originally developed in aviation and now widely applied in maritime operations, aims to reduce human error through effective communication, teamwork, and situational awareness (Salas et al., 2006). Its core competencies, verbal/non-verbal communication, decision-making, and adaptability, are highly dependent on ME proficiency. For this study, linking ME needs to CRM (e.g., VHF communication with VTS) ensures identified skills are behaviorally critical for on-board safety. CRM has become a core framework for maritime safety, yet its integration with ME needs analysis remains underdeveloped in prior research. Communication failures are widely recognized as a primary contributor to maritime accidents (Guo et al., 2025), with many such incidents traceable to inadequate ME proficiency in CRM-related scenarios, including shift handover procedures and emergency coordination efforts. For example, Yin et al. (2025) highlighted that miscommunication rooted in linguistic factors, specifically issues related to speech acts and lexical choices, was a key precursor to accidents, as well as to near-miss events and other high-risk operational situations. Among the linguistic dimensions studied, locutionary acts (centered on the clarity of expression) stood out as the most influential factor in communication breakdowns, followed by perlocutionary and illocutionary acts in terms of their impact on operational safety (Yin et al., 2025).

Reason’s (1990) “Swiss Cheese Model” identifies latent and active failures in systems that lead to accidents. Active failures, such as miscommunication during emergency procedures, are directly related to individual ME proficiency. Latent failures, such as inadequate ME training or poorly designed teaching materials, while latent failures (e.g., inadequate training) can be addressed through targeted needs analysis and curriculum reform. This framework highlights how linguistic gaps act as “holes” in the safety system, emphasizing the need for safety-critical ME skills.

In the development of language learning materials and curricula, particularly within the framework of ESP, conducting needs analysis is a methodological imperative (R. K. Sari et al., 2019). Basturkmen (2010) clarifies that needs analysis serves to identify target language use and requisite skills, thereby informing the determination and selection of appropriate learning materials in ESP. Numerous studies have applied needs analysis to ESP contexts across various disciplines, such as English education (Ekayati et al., 2020), physical education (Pranoto & Suprayogi, 2020), health sciences (Arroyyani et al., 2022), and informatics (R. K. Sari et al., 2019). The classical model proposed by Dudley-Evans and St John (1998) in ESP needs analysis provides a systematic approach to identifying the language skills required for specific professional contexts. This framework distinguishes between target situation needs and learning situation needs.

The core objective of ESP is to tailor courses to specific learning goals (Aslam & Irshad, 2025). In ME, target situation needs include communicating with port authorities, conducting safety drills, and documenting cargo operations, while learning situation needs encompass mastering technical vocabulary, understanding cross-cultural communication norms, and applying Standard Marine Communication Phrases (SMCP) in authentic scenarios, added ME-specific details to strengthen relevance. By integrating ESP needs analysis with behavioral science frameworks (e.g., Crew Resource Management, Reason’s model of human error), this study ensures that the identified ME skills are not only linguistically relevant but also behaviorally transferable to safety-critical on-board operations. Hutchinson and Waters (1987) emphasize that the primary goal of ESP courses is to enhance effective learning and optimize the achievement of profession-specific communicative competence, revised to clarify the original intent and align with ME’s practical orientation. Researchers such as Long (2005) and Kaewpet (2009) suggested using task-based method to needs analysis in teaching and learning.

Learner profiling, rooted in educational psychology and user experience research, integrates learners’ attributes, motivations, and needs (Park et al., 2015; Premlatha et al., 2016). In the context of ME education, this theory helps identify differences between exam-focused and work-focused learners. This distinction aligns with Self-Determination Theory (SDT) (Ryan & Deci, 2017), which emphasizes the role of autonomy, competence, and relatedness in motivation. SDT focuses on volitional behavior, distinguishing intrinsic motivation (engagement for pleasure) and internalization (actions based on inherent value), which explains the motivational differences between learner profiles.

With most international vessels employing multilingual crews, sociolinguistic factors in ME communication have become a key research focus. Numerous maritime accident reports have identified three recurring ME-related issues: incorrect SMCP use in distress messages, inadequate technical translation, and accent-related misunderstandings. A 2022 collision incident involving a Chinese vessel, where the crew’s failure to clearly describe a mechanical breakdown in English delayed tugboat assistance, resulted in significant cargo damage. This real-world evidence justifies the need to prioritize practical technical communication skills.

### 2.2. Teaching and Learning of ME

Relevant ME teaching studies highlight the value of situational and learner-centered approaches. Yashnikova (2022) verified that situational learning addresses seafarers’ practical language needs. By embedding critical thinking into language instruction, Rachman et al. (2024) provided an effective solution to the unique predicaments faced by maritime professionals, with positive implications for both communication efficiency and industry safety.

From the assessment perspective, integrating listening, speaking, reading, and writing to measure comprehensive communicative competence aligns with ESP principles of evaluating skills in professional contexts. On the learning side, L. I. Sari and Sari (2022) notes that weak general English proficiency and cultural barriers hinder ME acquisition, while Hellystia and Budiwaty (2023) stress aligning teaching with students’ perceptions in vocational settings. In response, researchers advocate contextually rooted methods. Agustina (2019) suggested that the implementation of Task-Based Language Teaching (TBLT) could serve as a viable pedagogical solution to address the ME learning challenges in vocational maritime education and training contexts, while Hofstede et al. (2010) call for integrating cultural awareness to address cross-cultural gaps.

### 2.3. Needs Analysis in Maritime English

Existing ME needs analysis has focused on non-English-speaking countries (e.g., Indonesia, the Philippines, Bangladesh). Dirgeyasa (2018) used questionnaire, documentary sheet, and interview to explore the ME learning materials for 48 Indonesian nautical students, emphasizing that ME resources must align with both regulatory requirements and on-board operational realities. Castro (2020) focused on communication needs, particularly in engine-deck interactions. The study pointed out a gap in the Philippine higher education curriculum, which lacks targeted training for communication in maritime contexts such as inter-ship and intra-ship exchanges, especially using the SMCP. Ahmmed et al. (2020) observed that basic communicative competence is a priority for Bangladeshi seafarers, but extends it to technical, scenario-specific communication.

Technological integration has emerged as a key trend in recent needs analysis. Batu et al. (2021) used questionnaires method to explore 318 students’ demand for ME learning apps, finding strong preference for digital resources that including explicit bilingual instructions and features like video, audio, and text. This reflects the growing need to incorporate digital literacy into ME needs assessment, addressing how learners engage with language tools beyond traditional textbooks. Herdawan et al. (2021) used questionnaire and interview method to investigate the ME needs of 19 Polimarin students. They suggested that syllabus revision is needed to improve the efficiency of English learning by meeting the students’ “necessities”, “lacks”, and “wants”.

Methodologically, mixed-methods approaches have become prevalent to ensure comprehensive needs capture. Rahman et al. (2022) combined questionnaires and interviews to determine cadet needs at Nusantara Maritime Academy Banjarmasin, confirming that ME course content must balance technical terminology, operational communication, and cross-cultural interaction.

Pedagogical innovation is another focal point. Kegalj et al. (2023) proposed a data-driven learning (DDL) approach for ME, using authentic linguistic data to foster student-centered, autonomous learning. Simanjuntak et al. (2024) further expanded this by examining new cadets’ literacy needs, finding that while technical proficiency is prioritized, socio-pragmatic skills (e.g., multinational crew interaction) are often neglected. Suprobo et al. (2025) explored integrating mental health literacy into Indonesian maritime vocational education, including ME courses, finding strong stakeholder support for multiliteracy frameworks that address language proficiency alongside well-being.

Critical comparison of existing research identifies three key gaps: (1) limited focus on Chinese seafarers’ unique needs (e.g., international port communication); (2) lack of integration between needs analysis and behavioral science frameworks (CRM, safety culture); (3) inadequate learner differentiation, with most studies ignoring motivational and experiential differences. This study addresses these gaps by focusing on Chinese pre-service students, integrating ESP theory with behavioral science (CRM, Reason’s model), and using mixed methods (questionnaires, interviews, learner profiling) to capture collective needs and individual differences.

### 2.4. Research Questions

Based on the gaps identified in the literature, this study aims to address the following research questions:

RQ1. What ME skills (including linguistic, behavioral, and sociolinguistic dimensions) do pre-service maritime students at Shanghai Maritime University need most to support safety-critical operations and CRM effectiveness?

RQ2. What are the types of learner profiles of ME students, and how are these types grounded in educational psychology and behavioral science theories?

RQ3. What are the differences in ME skill needs between learners with different profile types, and how can these differences inform differentiated teaching strategies aligned with ESP and behavioral science principles?

## 3. Methodology

This study adopts a mixed-methods approach, combining quantitative and qualitative data collection to comprehensively understand students’ ME needs. Mixed-methods research is particularly suitable for competency-based learning research (Poth et al., 2020). As it allows for both broad statistical analysis and in-depth exploration of behavioral and motivational factors. Among existing research tools, questionnaires are widely regarded as one of the most reliable tools for measuring the relationships between variables in needs analysis research, while interviews complement quantitative data by capturing contextual and subjective insights. The combination of questionnaires and interview methods enhances the validity and depth of needs analysis findings.

### 3.1. Research Design and Instruments

The study’s research design centers on three core components: questionnaire development, learner profiling construction, and interview protocol design.

#### 3.1.1. Questionnaire Design

The questionnaire was developed through a rigorous five-step adaptation process based on two core sources: the IMO Maritime English Model Course 3.17 and the validated ME needs analysis questionnaire by Ahmmed et al. (2020). First, the 14-theme, 68-item framework from Ahmmed et al. (2020) was adopted, covering core maritime scenarios such as radio communication, on-board communication, and safety operations. Second, combined with the core modules of IMO Model Course 3.17 (e.g., GMDSS communication, ISM Code safety management), 16 items were added to address the specific needs of Chinese seafarers (e.g., “negotiating with foreign employers”, “filling in port state inspection forms”, “applying for pilotage and tugboat assistance”), while 3 irrelevant items (e.g., “discussing marketing issues via VHF”) were deleted to ensure relevance to on-board operational safety. Third, all items were revised to align with the SMCP and Chinese maritime professional terminology standards (e.g., “Helm order” to “standard steering orders”, “Vessel Manoeuvring” to “berthing and unberthing procedures”). Fourth, content validity was verified by 4 experts (2 maritime English professors with 10+ years of ESP teaching experience and 2 senior captains with 20+ years of on-board operational experience), who evaluated each item for relevance, clarity, and core skill coverage. After four rounds of revisions, the final version included 15 themes and 84 items. Fifth, a pilot study was carried out with 30 pre-service maritime students. It confirmed good reliability and feasibility (average completion time = 15 min, item discrimination index = 0.32–0.67). The 15 thematic groupings are theoretically justified by the functional division of maritime operations (International Maritime Organization [IMO], 2015) and previous needs analysis studies (Ahmmed et al., 2020), covering all key communication scenarios from routine operations to emergency responses. The questionnaire is provided in File S1.

#### 3.1.2. Learner Profiling Construction

To address the research questions regarding learner characteristics, learner profiling theory (rooted in educational psychology and user experience research) was applied to analyze typological differences among ME learners. Drawing on Self-Determination Theory (Ryan & Deci, 2017) and learner characteristic, data from the questionnaire’s basic information section (e.g., academic year, internship experience, English proficiency, learning motivations) were used to construct learner profiles. The profile construction followed a four-stage framework: data collection, label extraction, system construction, and visualization, with statistical clustering analysis to ensure objectivity.

#### 3.1.3. Interview Protocol Design

To ensure the scientific and practical validity of the research results, 10 ME students with maritime internship experience were interviewed after questionnaire collection. The semi-structured interview protocol focused on three key areas: (1) learning objectives and motivations (aligned with self-determination theory), (2) perceived importance of ME skills for safety and operational performance (aligned with CRM and Reason’s model of human error), and (3) suggestions for improving ME courses (aligned with ESP course design principles). The interview questions were adapted from Ahmmed et al. (2020) and modified to fit the Chinese maritime context, with supplementary questions added during conversations to explore emerging themes. The interview questions are provided in File S2.

### 3.2. Participants

Participants were students and recent graduates from the Merchant Marine College at Shanghai Maritime University, a leading institution specializing in shipping, logistics, and marine research. To ensure sample representativeness, a stratified random sampling strategy combined with voluntary participation was adopted. Stratification was conducted by academic status (covering current students and recent graduates), with quotas proportional to the enrollment and graduate distribution of the college to minimize selection bias.

Researchers distributed online questionnaires via the school’s official academic management platform and WeChat groups, with clear explanations of the study purpose, data confidentiality commitments, and voluntary participation rights. Reminders were issued twice (on the 3rd and 6th days) to improve response rates. All participants signed an informed consent form.

This study collected 313 valid questionnaires, including 278 males (88.82%) and 35 females (11.18%), indirectly reflecting the gender distribution of the maritime industry. The average age of the respondents was 19.76 years (range: 18–26 years). The academic status distribution of the valid sample was as follows: freshmen (3.83%, n = 12), sophomores (48.56%, n = 152), juniors (31.63%, n = 99), seniors (5.43%, n = 17), recently graduated undergraduates (5.75%, n = 18), master’s students (4.47%, n = 14), and doctoral students (0.32%, n = 1). Approximately 30% of respondents (n = 94) had maritime internship experience. In terms of English proficiency, 83% passed CET-4 or CET-6, 3.52% passed TEM-4 or TEM-8, and 2.88% obtained ME qualifications or navigation-related certificates.

The sample characteristics align with the target population of pre-service Chinese maritime students, and the inclusion of students with varying internship experience and English proficiency allows for the construction of diverse learner profiles.

### 3.3. Data Collection Procedures

Data collection was conducted in two phases to ensure coherence and complementarity between quantitative and qualitative data. The questionnaire phase lasted 2 weeks (including two reminder rounds) to maximize response rates. Participants accessed the online questionnaire via a unique link, and data were automatically collated and cleaned. The interview phase began immediately after questionnaire collection. Ten participants were purposively selected to ensure representation across academic years, internship experience, and English proficiency levels. Interviews were conducted face-to-face or via video conferencing, audio-recorded, and transcribed verbatim (average duration: 15 min per interview). Participants were anonymized as Student 001 to Student 010, and member checking was conducted by sharing transcripts and thematic summaries with participants to confirm accuracy.

### 3.4. Data Analysis Methods

Data analysis was conducted in parallel for quantitative and qualitative data, with integration at the interpretation stage to address the research questions.

#### 3.4.1. Quantitative Data Analysis

The questionnaire adopted a 5-point Likert scale (1 = strongly disagree, 5 = strongly agree) to measure the level of need for each ME skill, with classification criteria as follows: 0–2.0 (Not needed), 2.1–3.0 (Less needed), 3.1–4.0 (Fairly needed), 4.1–4.5 (Needed), 4.51–5.0 (Highly needed).

To answer RQ1 (identifying highly needed ME skills), a one-sample *t*-test was conducted on each of the 84 items with a test value of 3 (neutral point), determining whether the average score was statistically significantly higher than “moderately needed”. To answer RQ3 (comparing skill needs between learner groups), independent sample *t*-tests were conducted for each item, with the Benjamini–Hochberg False Discovery Rate (FDR) procedure applied to control for Type I errors (84 items, critical *p* < 0.05). Effect sizes (Cohen’s d) were reported to indicate the magnitude of differences (d = 0.2 = small, d = 0.5 = medium, d = 0.8 = large).

To verify the structural validity of the 84-item questionnaire, exploratory factor analysis (EFA) was conducted using SPSS 26.0. The KMO value was 0.955, and Bartlett’s sphericity test yielded χ^2^ = 68263.043 (df = 3486, *p* < 0.001), indicating sufficient common factors for factor analysis. Principal Component Analysis (PCA) with varimax rotation extracted 15 common factors (eigenvalue > 1 criterion, scree plot inflection point) consistent with the theoretical thematic division. All 84 items had factor loadings > 0.6 on their corresponding factors, with no cross-loading (load difference > 0.2), confirming clear item-factor relationships. Convergent and discriminant validity were verified: the average variance extracted (AVE) for each of the 15 factors ranged from 0.52 to 0.76 (all > 0.5), and the square root of the AVE for each factor was greater than the correlation coefficient between that factor and other factors (reported in Supplementary Table S1 in File S3).The questionnaire was reviewed by 4 experts with high inter-rater reliability (Kappa = 0.89), ensuring comprehensive coverage of core ME skills for Chinese seafarers. Representative factor loadings of the questionnaire are reported in Supplementary Table S2 in File S3.

The internal consistency reliability of the questionnaire was tested using Cronbach’s α coefficient. The 84-item ME skill requirements questionnaire had a Cronbach’s α of 0.917, primarily due to two factors: (1) all items center on “maritime safety communication”, with interrelated skills across themes; (2) factor analysis confirms the 84 items can be divided into 15 distinct factors, each corresponding to a unique operational scenario. This high internal consistency suggests pre-service maritime students have a unified understanding of core ME skills required for on-board work, supporting the necessity of prioritizing these skills in curriculum design. Questionnaire reliability details are reported in Supplementary Table S3 (File S3).

#### 3.4.2. Qualitative Data Analysis

Interview data were analyzed using a mixed coding approach (deductive + inductive) based on a validated coding scheme, following Braun and Clarke’s (2006) six-step thematic analysis protocol. A deductive framework was constructed based on Self-Determination Theory, including three core categories (Learning Motivation, Behavioral Preference, Skill Priority) and subcodes. Validated by two independent experts (Kappa = 0.87, *p* < 0.001) prior to analysis, two maritime-specific subcodes were inductively added during transcript analysis: “SMCP application preference” (under Skill Priority) and “port operation communication anxiety” (under Behavioral Preference). Two trained coders independently coded 100% of transcripts using NVivo 12, with discrepancies resolved through consensus meetings with a third supervisor.

Inter-Rater Reliability (IRR) was confirmed with Cohen’s Kappa = 0.85 (categorical codes) and Intraclass Correlation Coefficient (ICC) = 0.89 (continuous variables), meeting rigorous qualitative research standards. Word frequency analysis quantified key themes: work-focused learners frequently mentioned “safety” (89 times), “internship” (76 times), and “VHF communication” (68 times), while exam-focused learners emphasized “exam” (92 times), “CET-4/6” (85 times), and “terminology” (69 times) (Supplementary Table S4 in File S3). These frequency counts were integrated with thematic coding to validate learner profiles. The validated coding scheme is reported in Supplementary Table S5 (File S3).

#### 3.4.3. Group Classification Criteria for “Exam-Focused” and “Work-Focused” Learners

To address concerns about subjective grouping, learner classification was operationalized via K-means clustering (K = 2) using 8 key indicators from the questionnaire: 3 items on learning motivation (5-point Likert scale), 2 items on internship experience (dichotomous/coded), and 3 items on skill demand preference (5-point Likert scale). The 8 indicators were standardized to eliminate dimensional differences, and the silhouette coefficient = 0.73 indicated good clustering quality. Independent samples *t*-tests confirmed significant differences in all indicators between the two groups (*p* < 0.001): exam-focused learners (n = 142) had higher scores on exam motivation, no/little internship experience, and preference for exam-focused skills; work-focused learners (n = 171) had higher scores on work motivation, long-term internship experience, and preference for practical skills. This classification was further validated by qualitative interview data, ensuring groups were formed based on objective statistical analysis and theoretical grounding rather than post hoc categorization. Clustering variable differences are reported in Supplementary Table S6 (File S3).

## 4. Results

Qualitative and quantitative data were analyzed in three main parts: identifying highly needed ME skills through quantitative responses, constructing learner profiles based on integrated quantitative and qualitative results, and analyzing differences in ME skill needs between profile types.

### 4.1. Quantitative Results from the ME Skills Questionnaire

The questionnaire covers 84 items across 15 topics, ranging from wireless communication to maritime document writing, including four basic language skills: listening, speaking, reading, and writing. Among the 84 skills, 24 were identified as “highly needed” by the respondents, with key results summarized (detailed results for all 84 items are provided in Supplementary Table S7 in File S3).

Radio communication skills were considered indispensable. Communicating with port authorities via VHF (M = 4.72, SD = 0.95, t (312) = 35.02, *p* < 0.001) was the most highly needed skill, followed by “responding to VTS (M = 4.66, SD = 0.93, t (312) = 34.57, *p* < 0.001) and sending/receiving distress messages (M = 4.55, SD = 1.04, t (312) = 30.27, *p* < 0.001). These skills directly align with CRM’s emphasis on effective information sharing and emergency response.

In the safety and security theme, seven items were classified as highly needed, including “Describing sea survival procedures” (M = 4.63, SD = 1.00, t (312) = 34.56, *p* < 0.001) and “Demonstrating safety drills” (M = 4.53, SD = 1.03, t (312) = 30.15, *p* < 0.001), highlighting the central role of safety-related communication in ME needs. Navigation-related skills such as “Understanding nautical charts and publications” (M = 4.62, t (312) = 33.57, *p* < 0.001) and “Giving directions on board” (M = 4.57, t (312) = 32.24, *p* < 0.001) demonstrated strong demand, indicating standardized operating protocols and navigation capabilities are key requirements for ME.

Four watchkeeping items were highly needed, such as “Understanding standing orders during shifts” (M = 4.63, SD = 0.98, t (312) = 34.17, *p* < 0.001) and “Describing duty rules” (M = 4.59, SD = 0.99, t (312) = 32.54, *p* < 0.001), reflecting the importance of standardized communication in routine operations. Significant differences were observed in translation skills: “Translating Chinese to English” (M = 4.26, SD = 1.26, t (312) = 18.24, *p* < 0.001) was rated as needed, while “Translating English to Chinese” (M = 2.52, SD = 1.15, t (312) = −11.63, *p* < 0.001) was rated as less needed. Notably, some highly needed skills showed large standard deviations (e.g., “Requesting medical assistance”: SD = 0.96), indicating variability in students’ perceptions, likely due to differences in internship experience (students with internships rated this skill higher, M = 4.89 vs. M = 4.51 for non-interns). This variability can be attributed to the contextual specificity of medical assistance scenarios: students with on-board internship experience have witnessed or participated in emergency medical drills (e.g., first aid communication with shore-based hospitals), which deepens their understanding of the skill’s criticality for crew safety. In contrast, non-intern students only encounter this skill through textbook descriptions, leading to weaker recognition of its practical relevance.

Social communication topics such as “Discussing cultural/religious beliefs” (M = 3.29, SD = 1.15, t (312) = 7.26, *p* < 0.001) and “Discussing leisure time” (M = 3.12, SD = 1.28, t (312) = 3.47, *p* = 0.0006) were rated as fairly needed, indicating lower priority compared to safety and operational skills.

**Figure 1:**
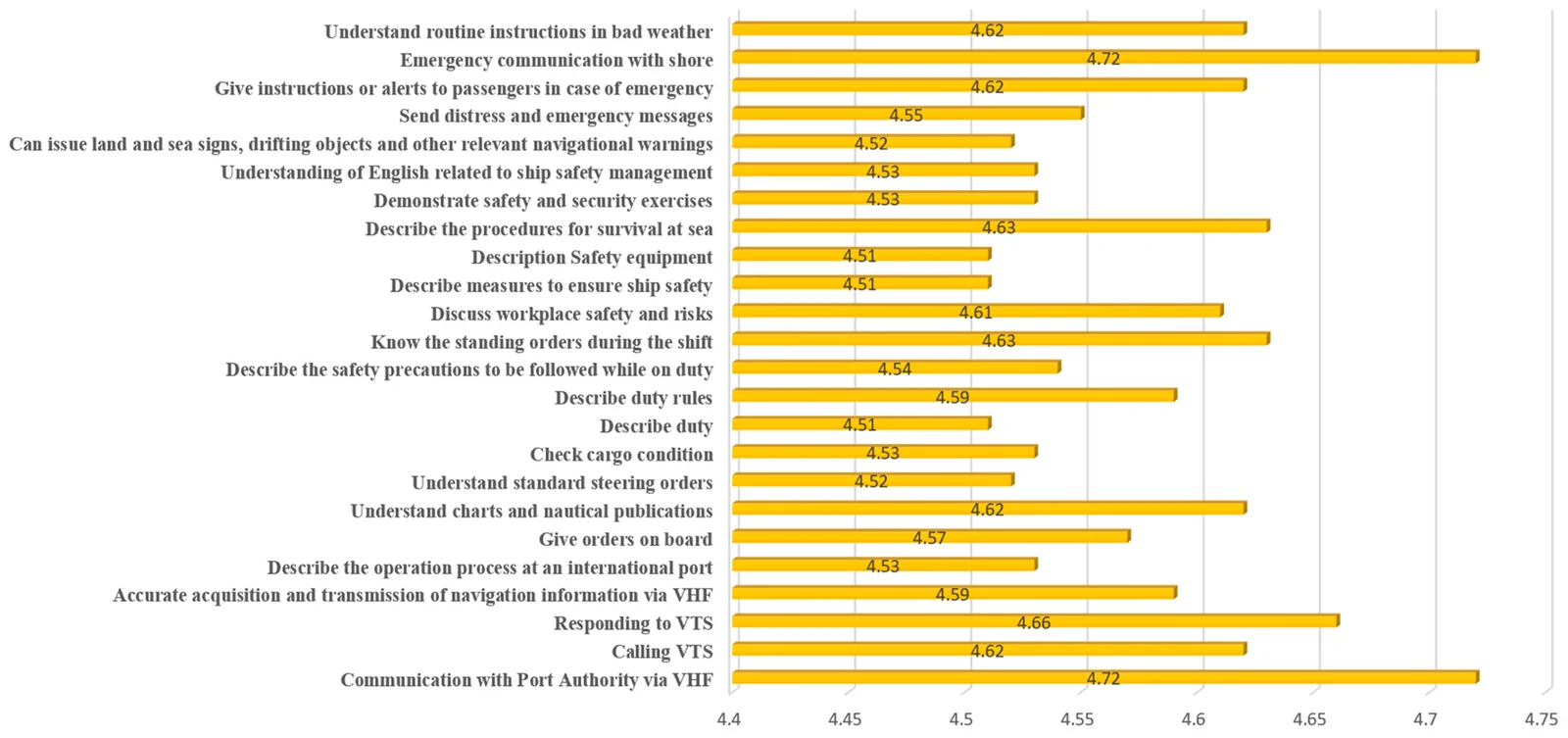
Distribution of 24 highly needed ME skills across 15 thematic categories. Note: Data are derived from 313 valid questionnaires, with “highly needed” defined as M ≥ 4.51 on a 5-point Likert scale.

Out of all 84 items, 24 are highly needed skills (as shown in Figure 1). Comprehensive analysis shows that the use of ME use is highly practical with particular emphasis on safety and emergency response.

The 24 highly needed skills showed a clear concentration trend (Figure 1): Safety and Security ranked first with 7 items (29.2%), followed by Radio Communication (5 items, 20.8%), Watchkeeping (4 items, 16.7%), and Emergency Situation and Medical Procedure (3 items, 12.5%). Cargo Operations and Weather each included 1 highly needed item. None of the 84 items were rated as unnecessary. This distribution suggests ME curriculum design should prioritize safety communication, radio operations, and emergency response.

### 4.2. Learner Profiling of ME Students

Based on questionnaire and interview data, two distinct learner profiles were identified, grounded in Self-Determination Theory and learner characteristics. The profiling framework helps locate curriculum goals, optimize teaching methods, and improve learning outcomes by identifying specific learning needs.

#### 4.2.1. Framework for Constructing ME Student Needs

**Figure 2:**
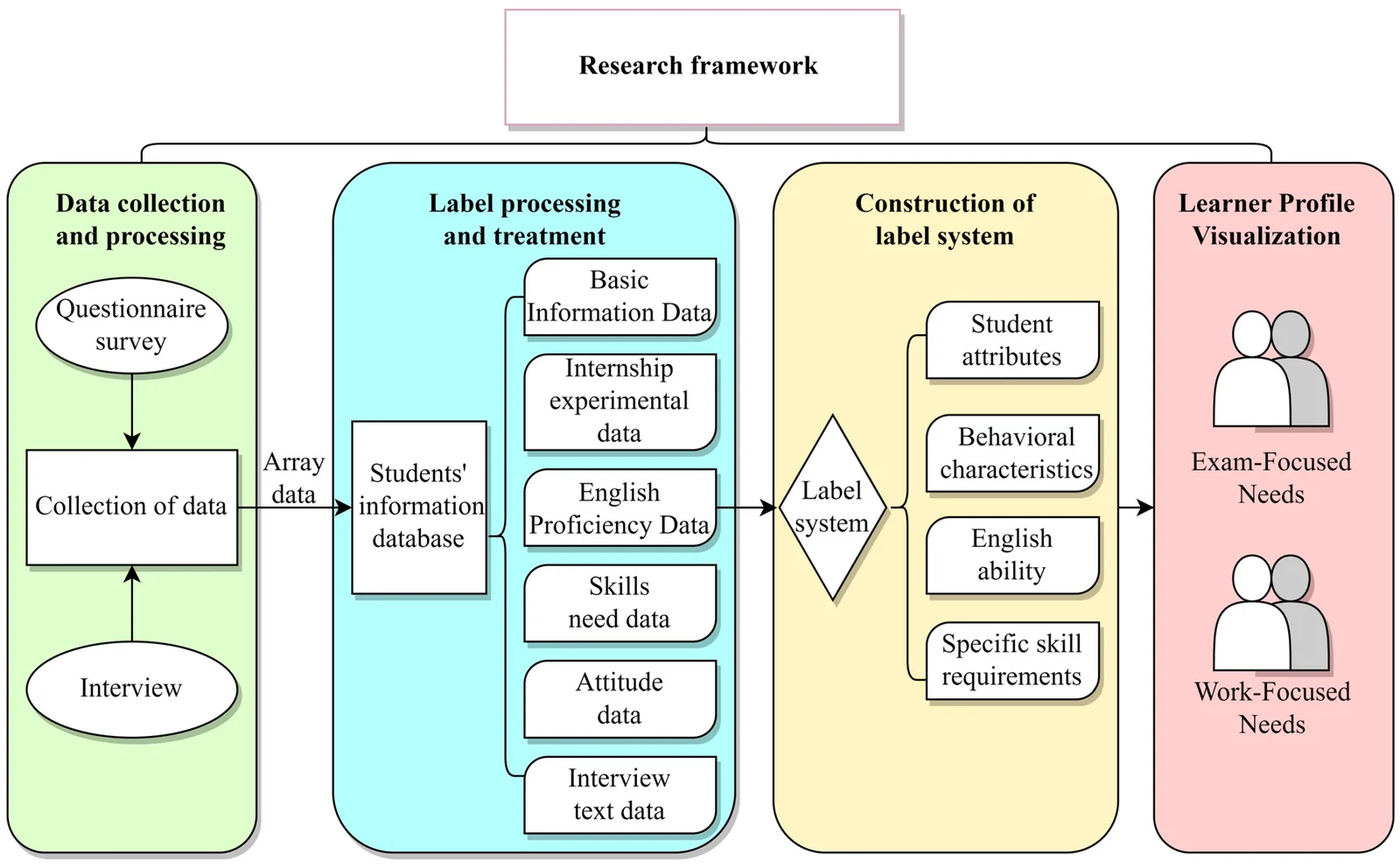
Framework for Constructing ME Learner Demand Profiles. Note: This framework integrates questionnaire and interview data to construct learner profiles through four sequential stages: Data Collection, Label Processing, Label System Construction, and Visualization.

The portrait research framework was shown in Figure 2.

As shown in Figure 2, the portrait research framework consists of four stages. (1) Step 1 is data collection and processing. Questionnaire data from Shanghai Maritime University students were sorted and classified to form a student information database. (2) Step 2 is label processing. The label system is theoretically grounded in Self-Determination Theory (Ryan & Deci, 2017) and the core professional identity characteristics, ensuring alignment with behavioral science frameworks. Invalid information was removed. The most frequent and relevant skill requirements were identified through word frequency analysis, focusing on students’ basic information, English proficiency, intern-ship experience, and skill needs. Open-ended interview questions were coded to obtain detailed insights. It specifically confirms the characteristics of user behavior and summarizes the data sets that can be used for processing. (3) The third step is the construction of the label system. Four aspects were used to create user portraits: student attributes, behavioral patterns, English proficiency, and skill importance. (4) The fourth step is the visualization of student portraits. Statistical icons and network diagrams were used to visually display student portraits of different roles and draw conclusions about student needs.

#### 4.2.2. Portrait Descriptions

Through comprehensive statistical analysis and structured data extraction from the questionnaire, this study developed a detailed portrait of ME learners. Student information was categorized and screened based on identified needs, which were further refined through word cloud analysis and text classification from interview data. After extracting the labels, the student’s needs portrait labels are summarized in four aspects. In combination with the student’s attributes, behavioral characteristics, English ability and skill importance needs, two distinct learner profiles were identified: exam-focused learners and work-focused learners. The two distinct learner profiles are detailed in Table 1.

As shown in Table 1, exam-focused learners (n = 142) are typically lower-grade students with limited ME exposure. They view ME as a required course, prioritize exam success over practical skills, and have little to no navigation-related internship experience. Their understanding of seafarers’ exam syllabus is limited, and they do not proactively set learning objectives. In terms of English proficiency, most have passed general English proficiency tests (e.g., CET-4/CET-6) but have not taken specialized ME or professional navigation exams. Their highly needed skills focus on radio communications, shipping manuals, freight operations, transportation details, watchkeeping, safety and security, emergency communications, translation and reading, and writing. This typology is further validated by qualitative interview data. Exam-focused learners frequently mentioned terms such as “CET-4/6” (85 times) and “exam preparation” (52 times) in interviews, which aligns with their high scores on exam motivation in quantitative clustering. Work-focused learners emphasized “safety” (89 times) and “internship” (76 times), consistent with their prioritization of practical operational skills in the questionnaire.

Work-focused learners (n = 171) are usually senior students with more ME course exposure. They recognize ME’s importance for future careers, have completed relevant internships (often longer durations), and are more aware of ME’s practical applications. They often hold advanced English certifications and navigation-related credentials. Their behavioral characteristics include actively seeking internships, focusing on all navigational communication skills, demonstrating a high degree of love for the course, and maintaining a proactive learning attitude. Their highly needed skills focus on radio communications, cargo operations, transport details, watchkeeping, safety and security, emergency communications, use of terminology, communication with external agencies, and shipboard communications.

#### 4.2.3. Differences in ME Skill Needs Between Profiles

To address the key issue of whether different learners have different views on ME skill requirements, a comparative analysis was conducted between exam-focused learners (n = 142) and work-focused learners (n = 171) student groups. This analysis conducted independent sample *t*-tests on each of the 84 maritime skills, comparing the average importance ratings between two identified learner groups.

The probability of obtaining false positive results (Type I errors) significantly increases when conducting multiple comparisons (84 independent sample *t*-tests). In order to control this risk and ensure the scientific validity of the findings, the error detection rate program of Benjamin Hochberg (B-H) was used in the article. Independent sample *t*-tests on the 84 ME skills (with Benjamini-Hochberg FDR correction) revealed 14 items with statistically significant differences (*p* < 0.05) between the two groups (Table 2). Work-focused learners rated practical operational skills significantly higher, with the largest differences observed in technically demanding areas. This advanced statistical method adjusts the significance threshold while maintaining statistical capability.

The most pronounced differences appear in mechanical equipment-related skills: “Describing main engine and propulsion systems” (work-focused = 4.71 vs. exam-focused = 3.76, t (311) = −7.74, *p* < 0.001, d = 0.92) and “Describing mechanical breakdown and repair” (work-focused = 4.58 vs. exam-focused = 3.62, t (311) = −7.44, *p* < 0.001, d = 0.88), indicating practical experience enhances understanding of core vessel technology maintenance.

In navigation operations, “Describing crew roles and routines about daily duties” showed a significant difference (work-focused = 4.71 vs. exam-focused = 4.25, t (311) = −7.72, *p* < 0.001, d = 0.89), reflecting work-focused learners’ greater emphasis on teamwork and daily management. Items such as “Describing procedures at international ports” (d = 0.81) and “Describing berthing and unberthing procedures” (d = 0.68) also showed medium effect sizes.

Notably, in safety skills, even with relatively low absolute ratings, work-focused learners showed higher need recognition for items like “Describing safety precautions while on duty” (d = 0.48) and “Describing sea survival procedures” (d = 0.47), suggesting practical experience enhances safety awareness.

The only exception was “Handling common issues and routine inspection communication,” which was rated slightly higher by exam-focused learners (exam-focused = 4.15 vs. work-focused = 3.91, t (311) = 2.13, *p* = 0.034, d = 0.24), potentially reflecting classroom emphasis on standardized procedures, while practical experience reveals the relative low priority of these routine tasks.

### 4.3. Interview Results

Following the quantitative analysis, 10 representative students (5 exam-focused, 5 work-focused) were selected for in-depth interviews to verify and enrich the findings on the two distinct learner profiles: exam-focused and work-focused. To maintain privacy, the students were anonymized and labeled as Students 001 to 010.

Exam-focused learners’ learning motivations centered on course credits and exam success: “I don’t have a detailed understanding of why the course was introduced. I haven’t reviewed the syllabus either, but it seems necessary to complete it for the credits.” (Student 003, Sophomore). They prioritized exam-related skills and desired easier exams: “I wish the exams were easier.” (Student 004, Junior). They viewed ME as “just another subject” and desired more engaging teaching: “The course could be more interesting and vivid.” (Student 002, Freshman).

Work-focused learners’ learning motivations were rooted in career relevance: “I’ve passed the seagoing seaman’s competency exam. With my internship experience in relevant organizations, I know ME is crucial not only for exams but also for future communication in the field.” (Student 009, Senior). They emphasized safety and practical skills aligned with CRM: “Safety is the most important thing, and we undergo extensive training for it before internships.” (Student 006, Senior). They advocated for more practical training: “ME courses should focus more on practical tasks. While theory and standard phrases are important, developing comprehensive communication skills is essential. In practical work, all the ME skills discussed are necessary. Expression and understanding form the foundation.” (Student 009, Senior).

Across both groups, there was consensus on the importance of safety-related ME skills, confirming alignment with behavioral science frameworks (CRM, Reason’s model). Word cloud analysis further visualized differences: work-focused learners’ key terms included “safety”, “internship”, “VHF”, “emergency”, while exam-focused learners’ key terms included “exam”, “CET-4/6”, “textbook”, “terminology” (Supplementary Figure S1 in File S3).

Regarding curriculum satisfaction, freshmen (exam-focused) felt ME’s practical significance was not well communicated: “As a new student, I only see this course as another subject. Its significance in practical life hasn’t been well communicated.” (Student 001). Senior students (work-focused) offered constructive feedback: “Classroom training is limited. To succeed, learners need to go beyond the curriculum, improving their overall abilities and learning related professional courses. If you want a career in navigation, personal effort is indispensable.” (Student 009).

In summary, the interviews confirmed the distinct needs and orientations of exam-focused and work-focused learners. These differences influenced their attitudes toward ME courses, their engagement in and outside the classroom, and their application of the language in real contexts.

## 5. Discussion

The present study’s findings identifying 24 highly needed ME skills and two distinct learner profiles offer critical insights into the intersection of linguistic needs, behavioral competencies, and maritime safety. Grounded in behavioral science frameworks (CRM, Reason’s human error model) and contemporary ESP theory, this discussion interprets results through theoretical lenses, contextualizes them within existing literature, and suggests actionable implications for curriculum design.

### 5.1. Theoretical Interpretation of Highly Needed Skills

The concentration of highly needed skills in safety and security (7 items) and radio communication (5 items) underscores the intrinsic link between ME proficiency and safety-critical behaviors, validating Reason’s (1990) “Swiss Cheese Model” of human error. The top-ranked skill VHF communication with port authorities (M = 4.72) directly maps to CRM’s core competency of effective information sharing, as miscommunication in this domain constitutes an “active failure” that can penetrate systemic defenses. This aligns with industry consensus identifying incorrect SMCP use in VHF exchanges as a leading cause of maritime accidents. It is consistent with the top-ranked highly needed skill in this study: ‘Communicating with port authority through VHF’. This convergence confirms that the identified ME skills are not only perceived by learners but also verified by the industry as safety-critical. The prominence of emergency-related skills such as distress message transmission, sea survival procedure description further reinforces that ME is not merely a linguistic tool but a behavioral competency that mitigates operational risk.

From an ESP perspective, the results validate the goal of contextualized competency mapping, which emphasizes integrating linguistic skills with professional behavioral requirements. The high demand for watchkeeping-related communication (4 items) reflects the target situation needs of routine maritime operations, while the low priority of English-to-Chinese translation aligns with the ESP principle of tailoring language instruction to the lingua franca nature of international maritime work. Notably, variability in responses to some skills (e.g., requesting medical assistance) highlights the influence of internship experience, students with on-board exposure rated practical skills significantly higher, underscoring the need to incorporate authentic situational learning into ME curricula. Notably, the high demand for China-specific skills such as “applying for pilotage and tugboat assistance” and “filling in port state inspection forms” distinguishes this study from prior needs analysis in Bangladesh (Ahmmed et al., 2020) and Indonesia (Dirgeyasa, 2018). Ahmmed et al. (2020) reported that Bangladeshi seafarers prioritize basic communication skills (e.g., daily crew interaction), while Dirgeyasa (2018) emphasized SMCP mastery for Indonesian cadets. This discrepancy reflects the unique operational context of Chinese seafarers, who frequently engage with international port authorities and cross-border logistics, highlighting the need for context-tailored ME curricula.

The low priority of English-to-Chinese translation contrasts sharply with industry reality: maritime safety research and incident analyses consistently identify “failure to articulate technical issues clearly in English” as a key contributing factor in maritime accidents. This discrepancy is likely driven by systemic curriculum gaps: most of interviewees (7 out of 10) reported no technical translation practice in ME courses, a proportion significantly higher than gaps in other skills (e.g., 23% for VHF communication, 19% for safety drills). This suggests that learners’ low perceived need reflects limited exposure to real-world translation scenarios rather than true irrelevance, emphasizing the need for curriculum reforms aligned with both learner perceptions and industry safety requirements (Ahmmed et al., 2020).

Contrary to the assumption that low ratings indicate irrelevance, the limited emphasis on English-to-Chinese translation likely stems from curriculum gaps (70% of interviewees reported no technical translation practice) rather than on-board utility, emphasizing the importance of needs analysis that accounts for both learner perceptions and industry realities. This finding alights with the previous research (Ahmmed et al., 2020).

### 5.2. Learner Profiling Based on Motivational and Behavioral Orientation

The identification of exam-focused and work-focused learners is theoretically grounded in Self-Determination Theory (Ryan & Deci, 2017). Exam-focused learners driven by extrinsic motivation prioritize textbook-related skills and exhibit passive learning behaviors. In contrast, work-focused learners motivated by intrinsic factors rooted in professional identity value practical skills (e.g., mechanical breakdown description, port procedure communication) with large effect sizes indicating meaningful behavioral differentiation. These differences are reinforced by thematic analysis of interview data and word cloud visualization of key terms (e.g., “safety” and “internship” for work-focused learners; “exam” and “CET-4/6” for exam-focused learners).

This typology extends prior ME needs analysis (Ahmmed et al., 2020) by linking learner characteristics to behavioral outcomes, addressing the gap in literature that separates linguistic needs from motivational drivers. For example, work-focused learners’ emphasis on cross-cultural communication (e.g., multinational crew interaction, M = 4.24) reflects sociolinguistic realities of modern maritime operations. In contrast, exam-focused learners’ relative neglect of these skills highlights the need to bridge extrinsic and intrinsic motivation through curriculum design. It should emphasize that linguistic accuracy alone is insufficient for safety; situational awareness and cultural adaptability are equally critical. This study extends previous ME needs analysis (Ahmmed et al., 2020; Dirgeyasa, 2018) by integrating behavioral science theories (SDT, CRM) to explain learner differences, rather than just describing skill needs and identifying China-specific needs (e.g., pilotage application, port state inspection document writing) overlooked in regional studies. Validating learner profiling with both quantitative clustering and qualitative coding, addressing methodological weaknesses in prior typology research.

For exam-focused learners, ESP course design principles suggest integrating exam preparation with practical applications to enhance intrinsic motivation. For example, linking textbook exercises to real-world maritime scenarios can help learners see the relevance of ME to their future careers. For work-focused learners, curriculum design should emphasize authentic tasks and CRM-aligned training, such as VR simulations of emergency communication or on-board teamwork exercises. These strategies address the needs of both learner types while ensuring alignment with behavioral science and ESP frameworks.

The significant differences in skill needs between the two groups (e.g., mechanical breakdown repair, crew role description) highlight the influence of internship experience on professional identity formation. As Hu et al. (2023) note, practical experience enhances learners’ understanding of the relevance of ME to their future roles, leading to more proactive learning behaviors. This suggests that early internship opportunities or simulated work experiences could help transform exam-focused learners into work-focused learners, improving the overall effectiveness of ME education.

From a behavioral science perspective, the findings extend beyond maritime education to safety communication research: (1) the link between internship experience and safety skill recognition reflects that ‘situational exposure’ is a key antecedent of safety communication competence; (2) the two learner profiles align with Self-Determination Theory’s distinction between extrinsic and intrinsic motivation, providing empirical evidence for motivation-based intervention in high-stakes professional education; (3) the concentration of high-need skills in safety and radio communication validates Reason’s “active failure” concept, suggesting that linguistic proficiency is a critical “human factor” in system safety. These insights bridge applied linguistics and behavioral science, offering a cross-disciplinary framework for safety-focused language education.

### 5.3. Practical Implications for Curriculum Design and Industry-Academia Collaboration

The heavy emphasis on examination systems (83% of participants prioritize CET-4/CET-6) creates a structural barrier to developing practical communication skills. Younger students (like Student 001) often view ME as “just another subject,” while those with industry exposure recognize its professional value. This suggests that early internship opportunities could transform learning motivation, as Hu et al. (2023) found in their study of professional identity formation. The relatively low priority of English-to-Chinese translation contrasts with Ahmmed et al.’s (2020) findings in Bangladesh, highlighting regional differences in ME needs.

The findings of this study offer actionable recommendations for ME curriculum design in maritime institutions with similar learner profiles. The study’s findings translate to concrete, evidence-based curriculum reforms tailored to learner profiles and aligned with behavioral science principles. For exam-focused learners: (1) Design “exam-practice integration” modules (e.g., linking terminology memorization to safety drill scenarios); (2) Provide targeted support for basic language skills (e.g., SMCP phrase drills aligned with exam questions); (3) Emphasize the connection between theoretical knowledge and practical application through case studies. For work-focused learners: (1) Offer advanced practical training (VR-based emergency communication simulations, on-board internships); (2) Strengthen complex situation handling and cross-cultural communication training (e.g., role-playing multinational crew interactions); (3) Invite industry experts to lead work-shops on technical communication (e.g., describing mechanical breakdowns).

Table 3 summarizes the differentiated curriculum recommendations tailored to the two learner profiles, with each strategy aligned with the identified high-demand skills and theoretical frameworks.

To further bridge the gap between classroom teaching and on-board practice, industry academia cooperation is very important. Based on experimental findings, this study proposes the following suggestions: (1) arrange structured, safety focused internships for sophomores (the largest sample group, 48.56%), as this group is mainly composed of exam focused learners with limited real-world ME use. These internships should integrate guided ME communication tasks, such as participating in VHF exchanges with port authorities or recording daily operations in English, to help students witness how textbook knowledge is translated into operational safety and crew coordination. (2) Invite senior captains with extensive onboard experience to lead scenario-based workshops centered around the high demand skills identified in this study, including mechanical fault communication and distress information transmission. These seminars should emphasize genuine behavioral expectations, such as precise and standardized language required for coordination with the VTS centers or multinational crew, directly addressing the practical communication gaps highlighted in interview data.

Assessment reforms are equally necessary to align evaluation with the study’s core findings and real-world maritime demands. Shift the focus of assessment from rote theoretical knowledge to practical communication competence in safety-critical contexts, incorporating simulation-based assessments (e.g., real-time VHF emergency distress message transmission, role-playing of shift handover communication that emphasizes CRM-aligned information sharing and teamwork). During the internship, workplace-based assessments were used to supplement class-room assessments. Supervisors rated students’ ability to apply ME skills in daily operations (e.g., cargo handling communication) and unexpected scenarios (e.g., requesting medical assistance), ensuring evaluations reflect the skills most valued by the industry.

To optimize the course content, priority should be given to 24 urgently needed skills, especially 7 safety and security skills and 5 most critical wireless communication skills, integrating them into core modules rather than peripheral content. Embedding safety culture training throughout the course, cultivating a “safety communication awareness”, and helping students recognize that language accuracy and clarity are not only technical skills, but also key guarantees for preventing human errors, as underestimated by the rational Swiss cheese model. Additionally, address the identified curriculum gap in technical translation by adding targeted practice aligned with maritime industry safety standards, which highlights “inability to translate vessel specific technical problems into clear English” as a recurring accident contributor. This training should focus on translating vessel-specific issues (e.g., mechanical malfunctions, cargo damage) into concise, standardized English, skills that 70% of interviewees reported lacking. These reforms, grounded in ESP course design principles and behavioral science frameworks, ensure that ME education not only enhances linguistic proficiency but also fosters the behavioral competencies essential for safe and effective maritime operations.

Kaewpet (2009, pp. 212–214) suggested the following principles for analyzing learner needs: “give first priority to communication needs, give equal importance to learning needs, take “context” into account, invite multiple perspectives, employ multiple data collection methods, treat needs analysis as on ongoing activity”.

ME course content should rebalance theoretical teaching and practical training. The construction of learning resources requires the development of intelligent navigation simulator laboratories, multilingual ship operation databases, and other teaching resources that combine reality and virtuality, and provide personalized learning paths based on learner type.

Industry-academia collaboration is critical to bridging the theory-practice gap: (1) Arrange structured internships for sophomores (the largest sample group, 48.56%) to expose exam-focused learners to real-world ME use; (2) Invite senior captains to lead scenario-based workshops focused on high-need skills (e.g., mechanical breakdown communication, distress message transmission) to infuse classroom learning with authentic behavioral expectations.

Assessment reforms are equally necessary. Shift assessment from theoretical knowledge to practical communication skills in safety-critical contexts, incorporating simulation-based assessments (e.g., emergency distress message transmission via VHF) and oral exams centered on CRM scenarios (e.g., shift handover communication). Include workplace-based assessments during internships to evaluate real-world application of ME skills.

To optimize curriculum content, prioritize the 24 highly needed skills (particularly safety and radio communication skills) as core teaching content, integrate safety culture training to cultivate “safety communication awareness”, and add technical translation practice aligned with incident report recommendations. These reforms align with ESP course design principles and behavioral science frameworks, ensuring ME education improves both linguistic proficiency and behavioral competencies for safe and effective maritime operations.

## 6. Conclusions

This study conducted a systematic needs analysis of pre-service maritime students at Shanghai Maritime University, adopting mixed-methods research to identify critical ME skills and explore learner-specific differences through theoretically grounded learner profiling. By combining data from 313 questionnaires and 10 in-depth interviews, the research identified 24 highly needed ME skills, concentrated in safety-critical domains (e.g., VHF communication, emergency procedures) and radio communication, and validated two distinct learner profiles (exam-focused and work-focused). These findings address key gaps in existing literature by linking linguistic needs to behavioral competencies, providing a rigorous, evidence-based foundation for ME curriculum reform.

The primacy of safety-related ME skills (7 out of 24 highly needed items) reinforces that ME proficiency is not merely a linguistic achievement but a behavioral competency directly influencing maritime safety. This aligns with Reason’s human error model and CRM principles, highlighting that effective ME communication mitigates active failures in high-stakes operations. The results further confirm the relevance of contemporary ESP theory, as the identified skills reflect both target situation needs (e.g., on-board operational communication) and learning situation needs (e.g., exam preparation for early-career students), validating the “contextualized competency mapping” framework for ME education.

The distinction between exam-focused and work-focused learners offers a deep understanding of motivational and behavioral differences in ME learning. Exam-focused learners prioritize exam-related skills driven by extrinsic motivation, while work-focused learners value applied skills reflecting intrinsic motivation and professional identity formation. This typology extends prior ME needs analysis by integrating motivational psychology, providing a theoretical basis for differentiated instruction.

Practically, the findings inform targeted curriculum reforms aligned with behavioral science and ESP principles. For exam-focused learners, integrating exam preparation with practical application bridges extrinsic and intrinsic motivation; for work-focused learners, CRM-aligned training strengthens skill transfer to real-world scenarios. Industry-academia collaboration including structured internships and expert-led workshops enhances ME instruction relevance, addressing the theory–practice gap.

This study has limitations related to sample representativeness: (1) Participants are exclusively from Shanghai Maritime University, which may limit generalizability to regional maritime colleges with different curriculum designs; (2) The sample is dominated by second-year students (48.56%) and male students (88.82%), underrepresenting female students and seniors with extensive internship experience. Future research should expand to multiple universities, balance demographic representation, and incorporate longitudinal tracking to explore how ME needs evolve as students transition to professional roles. Additionally, cross-cultural comparative studies could further contextualize findings within global maritime education.

Despite these limitations, this study enriches the discourse on ME education by integrating behavioral science and ESP theories, providing a theoretical foundation for ME needs analysis and curriculum design. The findings can guide curriculum developers and educators in designing more targeted and effective ME courses for pre-service maritime students in similar contexts, ultimately improving the safety and professional competitiveness of this group.

## Figures and Tables

**Table 1:** Description of ME Student Needs Portrait.

Terms	Portrait Description	
	Exam-Focused Learners (n = 142)	Work-Focused Learners (n = 171)
Student attributes	Lower grade student.	Senior or graduate students.
English ability	Passed the more common college English test.	Passed a higher level of English exam and even got a certificate in shipping.
Behavioral characteristics	There is no interest in internship or only a short period of internship practice.	Actively looking for internships and long-term internship practice.
	Passed an examination.	Achieve all kinds of communication in sailing work.
	Focus on the skills required for the test.	Pay attention to all the communicative skills needed in the process of navigation.
	The degree of love for the course is general.	High degree of love for the course.
	Learning attitude is relatively flat.	The learning attitude is more positive.
High needed skills focus	Radio communications, shipping manuals, freight operations, transportation details, watchkeeping, safety and security, emergency communications, translation and reading, writing.	Radio communications, cargo operations, transport details, watchkeeping, safety and security, emergency communications, use of terminology, communication with external agencies, shipboard communications.

**Table 2:** The ME demand values of exam-focused and work-focused learners after independent sample *t*-test and (B-H) test.

Item Number	Functions of Maritime Professions	Exam-Focused	Work-Focused	t-Value	*p* Value	Cohen’s d
22	Applying for pilotage, tugboat	4.01	4.49	−4.34	<0.001	0.50
23	Describing procedures at international ports	4.12	4.89	−6.67	<0.001	0.81
25	Describing crew roles and routines about daily duties	4.25	4.71	−7.72	<0.001	0.89
26	Describing berthing and unberthing procedures	3.85	4.52	−5.82	<0.001	0.68
29	Handle common issues and routine inspection communication	4.15	3.91	2.13	0.034	0.24
33	Describing main engine and propulsion	3.76	4.71	−7.74	<0.001	0.92
34	Understanding charts and nautical publications	4.32	4.90	−5.26	<0.001	0.68
40	Discussing cargo handling procedures	3.98	4.62	−5.32	<0.001	0.62
46	Describing the safety precautions to be followed while on duty	3.87	4.35	−4.08	<0.001	0.48
50	Describing safety equipment	4.26	4.71	−3.77	<0.001	0.44
51	Describing mechanical breakdown and repair	3.62	4.58	−7.44	<0.001	0.88
52	Describing procedures for survival at sea	4.16	4.63	−4.01	<0.001	0.47
66	Understanding meteorological information	4.18	4.97	−7.02	<0.001	0.87
82	Filling in the logbook of the voyage	4.35	4.87	−4.91	<0.001	0.59

Note: Independent samples *t*-test with Benjamini-Hochberg (B-H) FDR correction was applied; *p* < 0.05 indicates statistical significance.

**Table 3:** Summary of Differentiated Curriculum Recommendations for ME Courses.

Learner Profile	Core Objectives	Key Teaching Strategies	Targeted High-Demand Skills	Assessment Methods
Exam-Focused	1. Bridge exam preparation and practical relevance; enhance intrinsic motivation; 2. Consolidate basic linguistic skills to meet core exam requirements; 3. Establish initial awareness of safety communication.	1. Design “exam-practice integration” modules: Link SMCP phrase memorization to safety drill scenarios (e.g., adapt exam questions into VHF communication simulations); 2. Case-based teaching: Use simplified accident cases (e.g., minor hazards caused by misunderstood VHF instructions) to connect textbook knowledge to practical contexts; 3. Targeted skill training: Focus on exam-oriented high-frequency skills (terminology translation, nautical manual reading, standard document writing).	Radio communication (basic VHF communication), understanding of shipping manuals, standard steering orders, terminology translation and reading, basic safety and security skills.	1. Written tests (incorporate scenario-based questions, e.g., filling in standardized voyage logs); 2. Simulated oral exams (SMCP phrase application, basic safety instruction expression); 3. Assignments (linguistic analysis reports of accident cases).
Work-Focused	1. Strengthen skill transfer to real-world on-board operations; 2. Enhance core CRM competencies (team communication, emergency collaboration); 3. Address gaps in technical communication skills.	1. Immersive simulation training: VR-based simulations of emergency medical assistance, mechanical breakdown reporting, international port operations, etc.; 2. Cross-cultural collaboration training: Role-playing multinational crew interactions (e.g., communicating berthing procedures with foreign port authorities); 3. Industry expert workshops: Invite senior captains to explain practical skills such as “describing mechanical breakdowns” and “communicating crew role divisions.”	Radio communication (VTS response, distress message transmission), describing mechanical breakdown and repair, communicating international port procedures, describing crew roles and routines, requesting medical assistance, understanding nautical charts and publications.	1. Scenario-based practical assessments (e.g., VHF emergency communication drills, mechanical breakdown reporting simulations); 2. Cross-cultural communication tasks (e.g., writing operational instructions understandable to foreign crew); 3. Internship performance evaluation (on-board practical communication records).
Common for Both	1. Master safety-critical skills to meet industry safety requirements; 2. Proficiency in SMCP application in line with IMO standards; 3. Establish the awareness of “language-safety” connection.	1. Scenario-based core skill training: Focus on safety-related skills among the 24 highly needed skills (e.g., describing sea survival procedures, delivering safety drill instructions); 2. Standardized SMCP training: Mandate the use of standard phrases for key operational communication (e.g., berthing, shift handover); 3. Integration of safety culture: Strengthen communication safety awareness through accident case reviews (e.g., collision accidents caused by language misunderstandings).	Safety and Security (7 highly needed skills), Emergency Situation and Medical Procedure (3 highly needed skills), Watchkeeping (4 highly needed skills), standardized SMCP application.	1. Practical assessments in safety scenarios (e.g., emergency distress message transmission, delivering safety drill instructions); 2. SMCP application proficiency tests; 3. Language review reports of accident cases.

## Data Availability

The original contributions presented in this study are included in the article. Further inquiries can be directed to the corresponding author Xingrong Guo (xmguo@shmtu.edu.cn).

## Supplementary Materials

The following supporting information can be downloaded at: https://www.mdpi.com/article/10.3390/bs16010130/s1.

## Abbreviations

The following abbreviations are used in this manuscript:

ME	Maritime English
STCW	Standards of Training, Certification and Watchkeeping for Seafarers
TBLT	Task-Based Language Teaching
GME	General Maritime English
SMCP	Standard Maritime Communication Phrases
CRM	Crew Resource Management
ESP	English for Specific Purposes
VHF	Very High Frequency
VTS	Vessel Traffic Service
SDT	Self-Determination Theory
SCC	Safety Communication Competence
IRR	Inter-Rater Reliability
