# Correction: Guo et al. (2026). Enhancing Maritime Safety Through Needs Analysis: Identifying Critical English Communication Skills for Pre-Service Maritime Students in a Chinese University. *Behavioral Sciences*, *16*(1), 130

**DOI:** 10.3390/bs16030414

**Published:** 2026-03-12

**Authors:** Xingrong Guo, Mengyuan Zhen, Yiming Guo

**Affiliations:** 1 College of Foreign Languages, Shanghai Maritime University, 1550 Haigang Ave, Shanghai 201306, China; 202230810003@stu.shmtu.edu.cn; 2 School of Economics and Management, Shanghai Maritime University, 1550 Haigang Ave, Shanghai 201306, China

There was an error in the original publication (Guo et al., 2026). The original text incorrectly used the word “module” instead of the accurate term “questionnaire” in Section 3.4.1.

A correction has been made to one word (changed “module” to “questionnaire”) in 3.4.1. Quantitative Data Analysis, Paragraph 4:

The internal consistency reliability of the questionnaire was tested using Cronbach’s α coefficient. The 84-item ME skill requirements questionnaire had a Cronbach’s α of 0.917, primarily due to two factors: (1) all items center on “maritime safety communication”, with interrelated skills across themes; (2) factor analysis confirms the 84 items can be divided into 15 distinct factors, each corresponding to a unique operational scenario. This high internal consistency suggests pre-service maritime students have a unified understanding of core ME skills required for on-board work, supporting the necessity of prioritizing these skills in curriculum design. Questionnaire reliability details are reported in Supplementary Table S3 (File S3).

In the original publication, there was a mistake in Table 2 as published. The original Table 2 adopted an intermediate dataset, and the data have been revised to the finalized and verified research dataset. The corrected Table 2 appears below. The authors state that the scientific conclusions are unaffected. This correction was approved by the Academic Editor. The original publication has also been updated.

There was an error in the original publication. The original paragraphs 3–6 contained statistical data inconsistent with the finalized Table 2. The content has been revised to be fully consistent with the corrected Table 2.

A correction has been made to 4.2.3. Differences in ME Skill Needs Between Profiles, Paragraph 3, 4, 5 and 6:

The most pronounced differences appear in mechanical equipment-related skills: “Describing main engine and propulsion systems” (work-focused = 4.71 vs. exam-focused = 3.76, t (311) = −7.74, *p* < 0.001, d = 0.92) and “Describing mechanical breakdown and repair” (work-focused = 4.58 vs. exam-focused = 3.62, t (311) = −7.44, *p* < 0.001, d = 0.88), indicating practical experience enhances understanding of core vessel technology maintenance.

In navigation operations, “Describing crew roles and routines about daily duties” showed a significant difference (work-focused = 4.71 vs. exam-focused = 4.25, t (311) = −7.72, *p* < 0.001, d = 0.89), reflecting work-focused learners’ greater emphasis on teamwork and daily management. Items such as “Describing procedures at international ports” (d = 0.81) and “Describing berthing and unberthing procedures” (d = 0.68) also showed medium effect sizes.

Notably, in safety skills, even with relatively low absolute ratings, work-focused learners showed higher need recognition for items like “Describing safety precautions while on duty” (d = 0.48) and “Describing sea survival procedures” (d = 0.47), suggesting practical experience enhances safety awareness.

The only exception was “Handling common issues and routine inspection communication,” which was rated slightly higher by exam-focused learners (exam-focused = 4.15 vs. work-focused = 3.91, t (311) = 2.13, *p* = 0.034, d = 0.24), potentially reflecting classroom emphasis on standardized procedures, while practical experience reveals the relative low priority of these routine tasks.

There was a lack of clarity in the original publication. The phrase “addressing reviewers’ concerns about superficial connections to behavioral science” is redundant and should be deleted.

A correction has been made to **5.1 Theoretical Interpretation of Highly Needed Skills, Paragraph 1**:

The concentration of highly needed skills in safety and security (7 items) and radio communication (5 items) underscores the intrinsic link between ME proficiency and safety-critical behaviors, validating Reason’s (1990) “Swiss Cheese Model” of human error. The top-ranked skill VHF communication with port authorities (M = 4.72) directly maps to CRM’s core competency of effective information sharing, as miscommunication in this domain constitutes an “active failure” that can penetrate systemic defenses. This aligns with industry consensus identifying incorrect SMCP use in VHF exchanges as a leading cause of maritime accidents. It is consistent with the top-ranked highly needed skill in this study: ‘Communicating with port authority through VHF’. This convergence confirms that the identified ME skills are not only perceived by learners but also verified by the industry as safety-critical. The prominence of emergency-related skills such as distress message transmission, sea survival procedure description further reinforces that ME is not merely a linguistic tool but a behavioral competency that mitigates operational risk.

The authors state that the scientific conclusions are unaffected. This correction was approved by the Academic Editor. The original publication has also been updated.

## Figures and Tables

**Table 2:** The ME demand values of exam-focused and work-focused learners after independent sample *t*-test and (B-H) test.

Item Number	Functions of Maritime Professions	Exam-Focused	Work-Focused	t-Value	*p* Value	Cohen’s d
22	Applying for pilotage, tugboat	4.01	4.49	−4.34	<0.001	0.50
23	Describing procedures at international ports	4.12	4.89	−6.67	<0.001	0.81
25	Describing crew roles and routines about daily duties	4.25	4.71	−7.72	<0.001	0.89
26	Describing berthing and unberthing procedures	3.85	4.52	−5.82	<0.001	0.68
29	Handle common issues and routine inspection communication	4.15	3.91	2.13	0.034	0.24
33	Describing main engine and propulsion	3.76	4.71	−7.74	<0.001	0.92
34	Understanding charts and nautical publications	4.32	4.90	−5.26	<0.001	0.68
40	Discussing cargo handling procedures	3.98	4.62	−5.32	<0.001	0.62
46	Describing the safety precautions to be followed while on duty	3.87	4.35	−4.08	<0.001	0.48
50	Describing safety equipment	4.26	4.71	−3.77	<0.001	0.44
51	Describing mechanical breakdown and repair	3.62	4.58	−7.44	<0.001	0.88
52	Describing procedures for survival at sea	4.16	4.63	−4.01	<0.001	0.47
66	Understanding meteorological information	4.18	4.97	−7.02	<0.001	0.87
82	Filling in the logbook of the voyage	4.35	4.87	−4.91	<0.001	0.59

Note: Independent samples *t*-test with Benjamini-Hochberg (B-H) FDR correction was applied; *p* < 0.05 indicates statistical significance.
