# Supplementary material for: Enhancing Maritime Safety Through Needs Analysis: Identifying Critical English Communication Skills for Pre-Service Maritime Students in a Chinese University
Source: Behav Sci (Basel). 2026 Jan 16;16(1):130. doi: 10.3390/bs16010130 (PMC12837290; doi:10.3390/bs16010130)
Supplement: Supplementary file 1 [file behavsci-16-00130-s001.zip › behavsci-4026757-supplementary.pdf]

File S1. Maritime English Skills Questionnaire

Part I: Demographic and Background Information

Gender: ☐ Male ☐ Female
Academic year: ☐ Freshman ☐ Sophomore ☐ Junior ☐ Senior ☐ Master’s student ☐ Doctoral student
Maritime internship experience: ☐ None ☐ Short-term (⩽1 month) ☐ Long-term (>1 month)
Duration of maritime internship (if applicable): ______ months
English proficiency: ☐ Below CET-4 ☐ CET-4 ☐ CET-6 ☐ TEM-4 ☐ TEM-8 ☐ Maritime English certification (specify: ______) ☐ Other (specify: ______)
Primary goal for learning maritime English: ☐ Pass exams ☐ Prepare for future work ☐ Both ☐ Other (specify: ______)
Willingness to participate in follow-up interviews (optional): ☐ Yes ☐ No
Contact information (optional, only for follow-up interviews): ______

Part II: Questionnaire

Instructions

This questionnaire is adapted from Raju Ahmmed et al. (2020)'s maritime English needs analysis instrument and aligned with the International Maritime Organization (IMO) Maritime English Model Course 3.17 (2015). It aims to identify the maritime English skills critical for on-board work. Please rate the importance of each skill for your future maritime career using a 5-point Likert scale:
1 = Strongly Unnecessary (not required for on-board work)
2 = Unnecessary (rarely used in practical scenarios)
3 = Neutral (occasionally needed but not core)
4 = Necessary (regularly used in daily operations)
5 = Strongly Necessary (critical for safety and operational efficiency)
Your responses are anonymous and will only be used for academic research. Please answer truthfully based on your understanding of maritime work requirements.

Section 1: Radio Communication

1. Communicating with port authority through VHF
2. Calling upon Vessel Traffic Service (VTS)
3. Responding to Vessel Traffic Service (VTS)
4. Using VHF radio communications regarding bunkering
5. Taking and delivering navigational messages accurately via VHF radio
6. Naming positions on board
7. Ability to send and receive standard distress, emergency and safety messages using GMDSS

Section 2: On-board Communication

8. Communicating with multinational crew members
9. Understanding the cultural norms of different nationalities
10. Discussing cultural and religious beliefs
11. Discussing navigational routes and geographic locations
12. Sending emails
13. Asking for and giving personal data
14. Expressing personal likes and dislikes
15. Describing visitors on board
16. Discussing leisure time on board
17. Attending on-board meetings
18. Ordering food and discussing the food on board

Section 3: Communication with External Agencies

19. Negotiating with foreign employers
20. Confirming arrangements for joining a ship
21. Collaborating with other departments for joint services
22. Applying for pilotage and tugboat assistance

Section 4: Routine Works and Operations

23. Describing procedures at international ports
24. Giving directions on board
25. Describing crew roles and routines about daily duties
26. Describing berthing and unberthing procedures
27. Describing seafarer training
28. Reporting on shift change
29. Handling common issues and routine inspection communication

Section 5: Shipping Details

30. Naming types of vessels
31. Comparing vessel details
32. Describing changes in shipping, ship design and technology
33. Describing main engine and propulsion systems

Section 6: Shipping Manual

34. Understanding charts and nautical publications
35. Understanding ship operating instructions
36. Understanding standard steering orders
37. Understanding standard engine instructions
38. Understanding engineering drawings
39. Understanding instrument and equipment specifications

Section 7: Cargo Operations

40. Discussing cargo handling procedures
41. Checking supplies
42. Checking cargo number
43. Checking cargo condition

Section 8: Watchkeeping

44. Describing the duties of the shift
45. Describing duty rules
46. Describing the safety precautions to be followed while on duty
47. Knowing the standing orders during the shift

Section 9: Safety and Security

48. Discussing workplace safety and risks
49. Describing measures to ensure ship safety
50. Describing safety equipment
51. Describing mechanical breakdown and repair
52. Describing procedures for survival at sea
53. Demonstrating safety and security drills
54. Understanding English knowledge related to ship safety management
55. Sending navigation warnings about land/sea signs and drift objects

Section 10: Emergency Situation and Medical Procedure

56. Sending distress and urgency messages
57. Giving instructions to passengers in the event of an emergency
58. Understanding commands in emergency situations on board
59. Requesting medical assistance
60. Emergency communication with ashore

61. Providing psychological comfort to passengers after emergency events

Section 11: Reporting

62. Reporting incidents at sea
63. Reporting events from past voyages
64. Reporting damage caused by bad weather at sea
65. Reporting oil pollution ashore

Section 12: Weather

66. Understanding meteorological information
67. Understanding weather forecasts
68. Describing weather conditions
69. Understanding standing orders in bad weather

Section 13: Using Terminologies

70. Using maritime vocabulary
71. Using maritime terminologies
72. Using Standard Marine Communication Phrases (SMCP)
73. Understanding sentence and message patterns of SMCP

Section 14: Translation and Reading

74. Translating Chinese into English
75. Translating English into Chinese
76. Translating any other language into English/Chinese
77. Understanding international conventions/regulations in English
78. Understanding English abbreviations of maritime professional terms
79. Reading relevant materials in the General Operator knowledge area (English version, including SOLAS Convention terms, Radio regulations and related documents)
80. Reading English telegrams, telex and navigational warnings

Section 15: Official Document Writing

81. Filling in inbound and outbound reports
82. Filling in the logbook of the voyage
83. Filling out various declarations, maritime declarations, notifications, authorizations and other official documents
84. Filling in maritime accident reports, port state inspection forms, ship repair lists, engine logs, material lists, oil record books and other affair reports

File S2. Interview questions

First-level Themes	Second-level Questions
1. Learning purpose and motivation	1. Do you know the teaching objectives of Maritime English? What are your learning objectives?<br>2. Are you clear about the requirements of the seafarers' examination outline?<br>3. Why do you think Maritime English is important for your future career?
2. Learning behavior and attitude	4. Do you like Maritime English? Do you think it is necessary for the school to set up this course?<br>5. Will you make a study plan for Maritime English? If yes, how do you implement it?<br>6. What efforts have you made to improve your Maritime English proficiency?
3. Demand for specific ME skills	7. Which of the four language skills (listening, speaking, reading, writing) do you think are the most important for Maritime English? Why?<br>8. What ME skills do you think are most needed for safety and operational performance on ships?<br>9. How do you think ME skills can help reduce communication errors and improve safety?
4. Course improvement suggestions	10. Do you think the current ME course meets your learning needs? If not, what changes do you suggest?<br>11. What teaching methods or resources (e.g., simulations, internships, textbooks) do you think are most effective for learning ME?<br>12. How can the school better integrate practical maritime scenarios into ME teaching?

File S3: Supplementary Tables and Figures

Supplementary Table S1. Discriminant Validity Matrix (Extract)

Factor	Radio Communication	On-board Communication	Communication with External Agencies	Routine Works and Operations	Shipping Details
1	0.79	0.42	0.38	0.35	0.31
2	0.42	0.81	0.45	0.39	0.33
3	0.38	0.45	0.77	0.41	0.36
4	0.35	0.39	0.41	0.83	0.40
5	0.31	0.33	0.36	0.40	0.78

Note: Diagonal values are square roots of AVE; off-diagonal values are factor correlations.

Supplementary Table S2. Representative Factor Loadings of the Questionnaire

Item Number	Item Content	Factor Name	Factor Loading
1	Communicating with port authority through VHF	Radio Communication	0.892
8	Communicating with multinational crew members	On-board Communication	0.876
19	Negotiating with foreign employers	Communication with External Agencies	0.815
23	Describing procedures at international ports	Routine Works and Operations	0.851
33	Describing main engine and propulsion systems	Shipping Details	0.807
34	Understanding charts and nautical publications	Shipping Manual	0.827
43	Checking cargo condition	Cargo Operations	0.819
47	Knowing the standing orders during the shift	Watchkeeping	0.843
50	Describing safety equipment	Safety and Security	0.876
56	Sending distress and urgency messages	Emergency Situation and Medical Procedure	0.862
62	Reporting incidents at sea	Reporting	0.801
69	Understanding standing orders in bad weather	Weather	0.793
72	Using Standard Marine Communication Phrases (SMCP)	Using Terminologies	0.803
74	Translating Chinese into English	Translation and Reading	0.786
82	Filling in the logbook of the voyage	Official Document Writing	0.834

Supplementary Table S3. Questionnaire Reliability

**Questionnaire Module**	**Number of Items**	**Cronbach's α**	**Interpretation**
Essential information	6	0.623	Acceptable (⩾0.6)
Maritime English skill requirements	84	0.997	Very high
Total scale	90	0.917	Good (⩾0.8)

Supplementary Table S4. Keyword Frequency for Word Clouds

Work-Focused Learners		Exam-Focused Learners	
**Keyword**	**Frequency**	**Keyword**	**Frequency**
Safety	89	Exam	92
Internship	76	CET-4/6	85
VHF	68	Textbook	73
Emergency	62	Terminology	69
SMCP	57	Translation	58
Practical	53	Exam preparation	52
Safety drills	49	Vocabulary	48
Port	45	Course	43
Navigation	42	Credits	37
Communication	39	Reading comprehension	40
Crew	37	Exam skill	39
Vessel	35	Vocabulary memorization	38
Teamwork	33	Grammar exercise	36
Technical	31	Exam score	34
Drill	29	Course requirement	32
Navigation equipment	27	Study plan	30
Port authority	25	Textbook exercise	28
Distress message	23	Course Credit	26
Cross-cultural	21	Language test	24
Survival	19	Writing practice	22

Maintenance	17	Listening test	20
Pilotage	15	Speaking test	18
Bunkering	13	Terminology memorization	16
Logbook	11	Reading practice	14
Inspection	9	Grammar rule	12

Supplementary Table S5. Validated Coding Scheme for Interview Data

Core Category	Subcode	Definition	Example Quote
Learning Motivation	Exam-oriented motivation	Motivation driven by exams, certifications, or course credits	"I need to pass CET-6 and ME exams to graduate."
	Work-oriented motivation	Motivation driven by career relevance or practical application	"I must master VHF communication for future ship work."
Behavioral Preference	Active internship participation	Proactive participation in maritime-related internships (⩾1 month)	"I actively applied for a 3-month internship on a cargo ship."
	Passive internship participation	Involuntary or short-term internship experience (<1 month)	"I participated in a 2-week internship arranged by the school."
	Self-directed practical learning	Voluntary practice of ME skills (e.g., SMCP simulation, VHF drills)	"I practice VTS communication with classmates after class."
	Exam-focused learning	Learning activities centered on exam preparation (e.g., terminology memorization)	"I spend most of my time memorizing exam-related terms."
Skill Priority	Safety-critical skills	Preference for skills related to operational safety (e.g., emergency communication)	"Emergency distress messages are more important than translation."
	Exam-focused skills	Preference for skills frequently tested in exams (e.g., grammar, translation)	"I focus on terminology translation because it's a key exam item."
	Cross-cultural communication skills	Preference for skills related to multinational crew interaction	"I need to learn polite English expressions for communicating with foreign colleagues."
	SMCP application preference	Emphasis on using standardized maritime communication phrases	"SMCP phrases are essential for clear VHF communication."
	Port operation communication anxiety	Anxiety about communicating with port authorities or external agencies	"I'm nervous about applying for pilotage in English."

Supplementary Table S6. Clustering Variable Differences Between Two Learner Groups

Clustering Variables	Exam-focused Learners (M±SD)	Work-focused Learners (M±SD)	t-value	p value	Cohen's d	Effect Size Interpretation
Exam motivation score	4.21±0.68	2.89±0.75	18.72	<0.001	1.85	Large
Work motivation score	2.76±0.81	4.35±0.62	-21.34	<0.001	2.10	Large
Internship duration (coded)	0.32±0.51	1.78±0.63	-25.67	<0.001	2.53	Large
Preference for exam-focused skills	4.15±0.72	2.63±0.84	16.98	<0.001	1.67	Large
Preference for work-focused skills	2.83±0.79	4.42±0.65	-19.85	<0.001	1.96	Large

Supplementary Table S7. Quantitative Results from the ME Skills Questionnaire

Themes	*Functions of Martime Professions*	*M*	*Standard Deviation*	*t value*	*p value*	*Level of Need*
1.Radio Communication	1.Communicating to port authority through VHF	4.72	0.95	35.02	<0.001	Highly Needed
	2.Calling upon Vessel Traffic Service (VTS)	4.62	0.98	31.85	<0.001	Highly Needed
	3.Responding to Vessel Traffic Service (VTS)	4.66	0.93	34.57	<0.001	Highly Needed
	4.Using VHF radio communications regarding bunkering	4.25	1.05	23.29	<0.001	Needed
	5.Taking and delivering navigational messages accurately via VHF radio	4.59	0.96	33.18	<0.001	Highly Needed
	6.Naming positions on board	4.24	1.06	22.76	<0.001	Needed
	7.Ability to send and receive standard distress, emergency and safety messages using GMDSS	4.29	1.04	24.15	<0.001	Needed
2.On-board Communication	8.Communicating multi-nationalities	4.24	1.07	22.53	<0.001	Needed
	9.Understanding the cultural norms of different nationalities	4.2	1.08	21.42	<0.001	Needed
	10.Discussing cultural and religious beliefs	3.29	1.15	7.26	<0.001	Fairly Needed
	11.Discussing navigational routes and geographic locations	3.25	1.18	6.45	<0.001	Fairly Needed
	12.Sending emails	4.11	1.1	18.76	<0.001	Needed
	13.Asking for and giving personal data	3.03	1.25	0.89	0.374	Fairly Needed
	14.Expressing personal likes and dislikes	3.23	1.22	5.72	<0.001	Fairly Needed
	15.Describing visitors on board	3.21	1.23	5.38	<0.001	Fairly Needed
	16.Discussing leisure time on board	3.12	1.28	3.47	0.0006	Fairly Needed
	17.Attending on-board meetings	4.32	1.02	25.89	<0.001	Needed
	18.Order food and discuss the food on board	4.59	0.97	32.86	<0.001	Needed

3.Communication with external agencies	19.Negotiating with foreign employers	4.04	1.12	16.85	<0.001	Needed
	20.Confirming arrangements for joining a ship	4.2	1.1	21.23	<0.001	Needed
	21.Joint services with other departments	4.11	1.11	18.54	<0.001	Needed
	22.Applying for pilotage, tugboat	4.26	1.09	22.15	<0.001	Needed
4.Routine Works and Operations	23.Describing procedures at international ports	4.53	0.99	31.05	<0.001	Highly Needed
	24.Giving directions on- board	4.57	0.98	32.24	<0.001	Highly Needed
	25.Describing crew roles and routines about daily duties	4.27	1.08	22.53	<0.001	Needed
	26.Describing berthing and unberthing procedures	4.26	1.09	22.03	<0.001	Needed
	27.Describing seafarer training	4.24	1.1	21.47	<0.001	Needed
	28.Reporting on shift change	4.2	1.12	20.58	<0.001	Needed
	29.Handle common issues and routine inspection communication	4.25	1.09	22.37	<0.001	Needed
5.Shipping details	30.Particulars Naming types of vessel	4.2	1.13	20.15	<0.001	Needed
	31.Comparing vessel details	4.26	1.11	21.76	<0.001	Needed
	32.Describing changes in shipping, ship design and technology	4.26	1.12	21.35	<0.001	Needed
	33.Describing main engine and propulsion	4.21	1.13	20.18	<0.001	Needed
6.Shipping manual	34.Understanding charts and nautical publications	4.62	0.96	33.57	<0.001	Highly Needed
	35.Understanding ship operating instructions	4.2	1.14	19.76	<0.001	Needed
	36.Understanding standard steering orders	4.52	0.97	32.04	<0.001	Highly Needed
	37.Understanding standard engine instructions	4.24	1.15	20.53	<0.001	Needed
	38.Understanding engineering drawings	4.27	1.14	21.15	<0.001	Needed
	39.Understanding instrument and equipment specifications	4.29	1.13	22.07	<0.001	Needed

7. Cargo Operations	40.Discussing cargo handling procedures	4.28	1.14	21.65	<0.001	Needed
	41.Checking supplies	4.22	1.16	19.98	<0.001	Needed
	42.Checking cargo number	4.27	1.15	20.87	<0.001	Needed
	43.Checking cargo condition	4.53	1	30.76	<0.001	Highly Needed
8.Watchkeeping	44.Describing the duties of the shift	4.51	1.01	29.98	<0.001	Highly Needed
	45.Describing duty rules	4.59	0.99	32.54	<0.001	Highly Needed
	46.Describing the safety precautions to be followed while on duty	4.54	1.01	31.25	<0.001	Highly Needed
	47.Knowing the standing orders during the shift	4.63	0.98	34.17	<0.001	Highly Needed
9.Safety and security	48.Discussing workplace safety and risks	4.61	0.99	32.86	<0.001	Highly Needed
	49.Describing measures to ensure ship safety	4.51	1.02	29.27	<0.001	Highly Needed
	50.Describing safety equipment	4.51	1.03	28.85	<0.001	Highly Needed
	51.Describing mechanical breakdown and repair	4.24	1.2	18.97	<0.001	Needed
	52.Describing procedures for survival at sea	4.63	1	34.56	<0.001	Highly Needed
	53.Demonstrating safety and security drills	4.53	1.03	30.15	<0.001	Highly Needed
	54.Understanding English knowledge related to ship safety management	4.53	1.04	29.76	<0.001	Highly Needed
	55.Sending land and sea signs, drift objects and other related navigation warnings	4.52	1.05	29.08	<0.001	Highly Needed
10.Emergency Situation and Medical Procedure	56.Sending distress and urgency messages	4.55	1.04	30.27	<0.001	Highly Needed
	57.Giving instructions to passengers in the event of an emergency	4.62	1.01	33.25	<0.001	Highly Needed
	58.Understanding commands in emergency situations on board	4.27	1.22	19.65	<0.001	Needed
	59.Requesting medical assistance	4.72	0.96	34.75	<0.001	Highly Needed

	60.Emergency communication with ashore	4.25	1.21	19.87	<0.001	Needed
	61.Psychological comfort to passengers after emergency events	4.28	1.18	21.05	<0.001	Needed
11.Reporting	62.Reporting incidents at sea	4.24	1.23	18.76	<0.001	Needed
	63.Reporting events from past voyages	4.26	1.2	19.98	<0.001	Needed
	64.Reporting damage caused by bad weather at sea	4.26	1.21	19.65	<0.001	Needed
	65.Reporting oil pollution ashore	4.26	1.22	19.32	<0.001	Needed
12.Weather	66.Understanding meteorological information	4.28	1.2	20.87	<0.001	Needed
	67.Understanding weather forecasts	4.27	1.23	19.25	<0.001	Needed
	68.Describing weather conditions	4.24	1.24	18.43	<0.001	Needed
	69.Understanding standing order in bad weather	4.62	1.05	31.57	<0.001	Highly Needed
13.Using Terminologies	70.Using Maritime Vocabulary	4.24	1.25	18.12	<0.001	Needed
	71.Using Maritime Terminologies	4.24	1.26	17.83	<0.001	Needed
	72.Using Standard Marine Communication Phrases (SMCP)	4.12	1.28	15.67	<0.001	Needed
	73.Understanding sentence and message patterns of SMCP	4.25	1.27	17.95	<0.001	Needed
14.Translation and Reading	74.Translating Chinese into English	4.26	1.26	18.24	<0.001	Needed
	75.Translating English into Chinese	2.52	1.15	-11.63	<0.001	Less Needed
	76.Translating any other language into English/Chinese	2.46	1.18	-12.75	<0.001	Less Needed
	77.Understanding international conventions/regulations in English	2.92	1.25	-1.87	0.062	Less Needed
	78.Understanding English abbreviations of maritime professional English	2.28	1.12	-16.58	<0.001	Less Needed

15. Official document writing	79.Reading relevant materials in the General Operator knowledge area (English version) (including the terms of the SOLAS Convention, Radio regulations and related documents)	4.28	1.22	20.15	<0.001	Needed
	80.Reading English telegrams, telex and navigational warnings	4.3	1.2	21.37	<0.001	Needed
	81.Filling in the inbound and outbound reports	4.24	1.27	17.83	<0.001	Needed
	82.Filling in the logbook of the voyage	4.26	1.25	18.56	<0.001	Needed
	83.Filling out all kinds of declarations, maritime declarations, notifications and authorizations, various reports and other official documents.	4.27	1.24	18.97	<0.001	Needed
	84.Filling in the maritime accident report, port state inspection, ship repair list, engine log, material list, oil record book and other affairs report.	4.26	1.26	18.24	<0.001	Needed

Supplementary Fig.S1 Word Clouds of Interview Transcripts by Learner Type

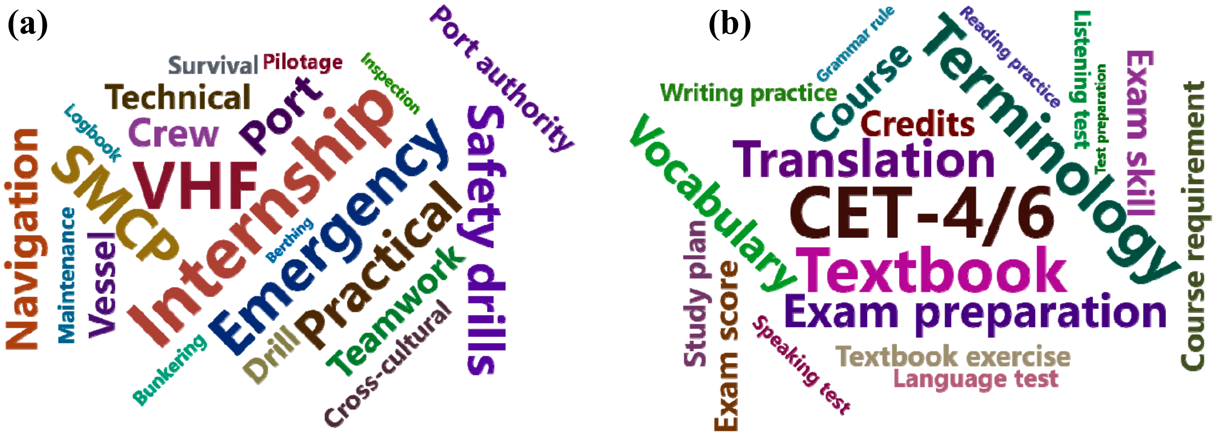

Supplementary Fig. S1 Word clouds of interview transcripts by learner type. (a) Work-Focused Learners (key terms: safety, internship, VHF); (b) Exam-Focused Learners (key terms: exam, CET-4/6, textbook). Note: Font size is proportional to word frequency (frequency data in Supplementary Table S4).
